# The nucleolus: a central response hub for the stressors that drive cancer progression

**DOI:** 10.1007/s00018-019-03231-0

**Published:** 2019-07-23

**Authors:** Shannon E. Weeks, Brandon J. Metge, Rajeev S. Samant

**Affiliations:** 1 Department of Pathology, University of Alabama at Birmingham, WTI 320E, 1824 6th Ave South, Birmingham, AL 35233 USA; 2 O′Neal Comprehensive Cancer Center, University of Alabama at Birmingham, Birmingham, AL USA

**Keywords:** Nucleolus, Cancer, Pol I, rRNA, Ribosome

## Abstract

The nucleolus is a sub-nuclear body known primarily for its role in ribosome biogenesis. Increased number and/or size of nucleoli have historically been used by pathologists as a prognostic indicator of cancerous lesions. This increase in nucleolar number and/or size is classically attributed to the increased need for protein synthesis in cancer cells. However, evidences suggest that the nucleolus plays critical roles in many cellular functions in both normal cell biology and disease pathologies, including cancer. As new functions of the nucleolus are elucidated, there is mounting evidence to support the role of the nucleolus in regulating additional cellular functions, particularly response to cellular stressors, maintenance of genome stability, and DNA damage repair, as well as the regulation of gene expression and biogenesis of several ribonucleoproteins. This review highlights the central role of the nucleolus in carcinogenesis and cancer progression and discusses how cancer cells may become “addicted” to nucleolar functions.

## Background

The nucleolus is a non-membrane bound, sub-nuclear body that forms around tandem arrays of ribosomal gene repeats, known as nucleolar organizing regions (NORs) [1], on acrosomal chromosomes (chromosome 13, 14, 15, 21, and 22). Although the first person to observe the nucleolus is unknown, there are records of Felice Fontana making note of its presence in eel skin in 1781 [2]. The nucleolus is known to be formed by “the act of building ribosomes” [3]. The nucleolus itself consists of four regions that all have different functions. These are the fibrillar center (FC), dense fibrillar component (DFC), the granular component (GC), and the perinucleolar compartment (PNC) [4, 5]. The GC is the largest of these sub-compartments, surrounding the DFC and the FC, and contains immature ribosomes. The DFC surrounds the FC. The FC contains the sites of rDNA that are actively being transcribed and, the DFC contains proteins such as fibrillarin that are important for rRNA processing [6]. The PNC is associated with, but structurally distinct from, the nucleolus. The PNC contains large amounts of the heterogeneous nuclear ribonucleoprotein (hnRNP) complex, many RNA-binding proteins, as well as RNA polymerase III transcripts [7, 8].

Ribosome biogenesis occurs primarily in the nucleolus and is concluded in the nucleoplasm and cytoplasm [9]. rDNA is transcribed in the nucleolus by a multiprotein complex consisting of RNA polymerase I (Pol I), the rDNA transcription factor Rrn3, Selectivity factor 1 (SL1), and Upstream-Binding Factor (UBF) which assemble at the rDNA promoter and initiate transcription. After initiation, the transcript is elongated by Pol I [10]. The resulting product of rDNA transcription is the 47S rRNA precursor (pre-rRNA) which then undergoes additional processing to produce the mature 18S, 5.8S, and 28S rRNA [9]. The 5S rRNA is transcribed in the nucleoplasm by Pol III and is later imported into the nucleolus where it will become part of the large ribosomal subunit [11]. The 5S, 5.8S, 18S, and 28S rRNAs are then assembled with ribosomal proteins (RPs) in the GC to form the large and small ribosomal subunits. The large 60S subunit is made up of the 28S, 5.8S, and 5S RNAs, in addition to 47 ribosomal proteins (RPLs). The small 40S subunit contains only the 18S RNA and 32 ribosomal proteins (RPSs) [12]. The large and small ribosomal subunits then translocate into the cytoplasm, where they undergo additional processing to produce the mature ribosome [13]. The production rate of ribosomes is dependent on Pol I activity, making Pol I activity an excellent tool for measuring ribosome biogenesis [14].

## Involvement of the nucleolus in various disease pathologies (Table 1)

Alterations in nucleolar function and ribosome biogenesis have a noticeable association with several major disease pathologies such as neurological diseases (viz. Parkinson’s disease (PD) and Huntington’s disease) and cardiovascular pathophysiologies (viz. ischemia, heart failure, myocardial infarction and cardiomyocyte hypertrophy).

**Table 1:** The role of the nucleolus in disease pathologies

Disease	Nucleolar involvement
Parkinson’s disease	Altered nucleolar morphology Nucleolin interacts with mutated RNAs resulting in reduction of rRNA transcription Increase in nucleolin found to be neuroprotective
Cardiac hypertrophy	Increased AgNOR in patients with ischemic heart disease Increase in NPM1 and NS levels in hypertrophic hearts Transcriptional and genotoxic stress leads to NPM1 and NS translocating out of the nucleolus
Dyskeratosis congenita	Caused by mutation on DKC1 gene for the nucleolar protein dyskerin Dyskerin protein is crucial for proper folding of rRNA and ribosome biogenesis
Diamond Blackfan anemia	Caused by mutation in ribosomal protein RPS19 Results in increased likelihood of developing hematopoietic malignancies
Myelodysplastic syndrome (MDS)	In MDS cells, ribosomal protein RPL23 is found to be a negative regulator of apoptosis Increased levels of RPL23 are associated with an increased likelihood of developing acute myeloid leukemia

In PD patients, dopaminergic neurons specifically have been found to have altered nucleolar morphology and functionality [15]. Nucleolin (NCL), a known nucleolar phosphoprotein involved in rRNA transcription, plays a pathogenic role in neurodegenerative diseases such as PD. In PD, nucleolin causes a reduction in rRNA transcription by interacting with mutated RNAs commonly found in polyglutaminopathies leading to an increase in nucleolar stress [16, 17]. Additionally, in the substantia nigra, part of the brain involved in controlling movement, nucleolin is found to play a role in the neurotoxic effects of retinone, a compound used to induce PD-like symptoms [18]. Furthermore, the overexpression of nucleolin showed a neuroprotective effect in cellular models of PD [18], clearly highlighting the importance of nucleolin and the nucleolus in neurological disorders such as PD.

Cardiovascular pathophysiologies show involvement of several key nucleolar proteins, including nucleophosmin (NPM1) and nucleostemin (NS). Levels of NPM1 were increased in the hearts of mice that have suffered heart attacks or have cardiac hypertrophy due to chronic high blood pressure [19]. Additionally, in canine models of obesity-induced hypertension, NPM1 expression increases twofold in the atria [20]. Transcriptional and genotoxic stresses cause both NPM1 and NS, to delocalize from the nucleolus, indicating an early stress response in cardiac myocytes [19]. NS has been found to be important in cardiac development with its expression declining after birth. However, NS levels are elevated in cardiac progenitor cells and cardio-myocytes in the border zone of ischemic hearts. Further studies showed that patients with cardiac hypertrophy caused by chronic high blood pressure have increased levels of NS. These findings clearly highlight the role of both NS and NPM1 in proliferative signaling in the heart [21]. In addition to various nucleolar proteins, NOR plays an important role in cardiac function and pathology. In hypertensive hearts, a positive correlation is seen between the activity of cardiac NORs, myocardial weight, maximal diastolic pressure, and left ventricular wall thickness [22]. Furthermore, increased AgNORs (the argyrophilic nucleolar organizing regions) were observed in specimens from patients with ischemic heart disease that was not complicated by heart failure compared to healthy controls. However, in patients with progressive ischemic heart disease there was a negative association between AgNOR and the level of heart failure severity. [23]. These findings further support the hypothesis that the nucleolus plays a crucial role in cardiac pathology as well as growth and development.

The nucleolus is also known to play a role in viral infection. While viral proteins often localize to the nucleolus, viral infections can cause important changes in the localization of various host nucleolar proteins [24]. These alterations, depending on the context, aid or inhibit viral replication. Due to the multifunctional nature of the nucleolus, these alterations are not entirely unexpected. In the case of Semliki Forest virus, the protein nsP2, a multifunctional protein required for viral replication, localizes primarily in the nucleus and nucleoli [25]. Unfortunately, no additional studies have been done to further elucidate the role of this viral protein in the nucleolus. Additionally, the West Nile Virus capsid proteins are found to bind the nucleolar protein DDX56, an RNA helicase protein, resulting in the relocation of DDX56 to the cytoplasm where it is involved in the post-replicative assembly of virions. It was observed that virions from DDX56 depleted cells are 100 times less infectious than those virions produced in DDX56-expressing cells [26]. Nucleolin is also involved in the infection process of numerous RNA and DNA viruses including hepatitis C virus, influenza A virus, adeno-associated virus type 2, as well as human papillomavirus. Nucleolin is involved in a variety of critical processes in the viral life cycle. It can bind directly or indirectly to viral factors associated with the viral life cycle and thereby plays a role in virus-associated pathogenesis [27–30].

In addition to the nucleolus’s involvement in multiple pathologies discussed so far, alterations in nucleolar proteins and functions have also been shown to be linked to an increased likelihood of developing cancer. In subsequent sections, we will focus on the involvement of the nucleolus and its components in cancer.

## The nucleolus: a key sensor of stress (Table 2)

Cellular stress is a common occurrence in the life of every cell, and, therefore, they must be able to sense and respond to these alterations in homeostasis. Due to the fact that ribosome biogenesis is one of the most energy expensive processes in the cell, response to cellular stress is most commonly achieved by downregulating the synthesis of rRNA and ribosome biogenesis [31]. This clearly makes the nucleolus, the site of ribosome biogenesis, a key player and central hub in sensing and responding to cellular stress. In addition to the response to the DNA damage (discussed in the following section), the nucleolus is involved in the stress response for several different types of physiological stressors including hypoxia, pH fluctuation, and redox stress.

**Table 2:** The role of nucleolar proteins in cellular stress response

Nucleolar protein	Involvement in stress response
BLM and WRN	Pol I interactors involved in DNA damage repair KD can result in decrease in pre-rRNA transcription
NPM1	Disrupts p53–HDM1 interaction when translocates out of nucleolus Involved in DNA damage repair through BER pathway
VHL	HIF stabilization under normoxic conditions when VHL is sequestered to the nucleolus
NCL	KD results in increased radiosensitivity Involved in viral replication
DDX56	Involved in viral replication and infection

Hypoxia and acidosis can be caused by a variety of different pathological and physiological conditions, including tumor development, muscle stress, and ischemic disorders. A key protein in cells’ ability to respond to hypoxia is hypoxia-inducible factor (HIF), a transcription factor that activates a variety of genes known to be involved in tumor vascularization, oxygen homeostasis, and ischemic preconditioning [32]. HIF is stabilized by hypoxia, and in the presence of oxygen, is degraded by the tumor suppressor VHL (von Hippel-Lindau) [33]. A study by Mekhail et al. found that hypoxia induction or normoxic acidosis can neutralize the VHL by triggering its nucleolar sequestration. When VHL is sequestered to the nucleolus, HIF is not degraded in normoxic conditions and is able to activate its target genes and drive tumor progression [34]. These findings lend further support to the nucleolus as a hub for nucleolar stress response.

In addition to sensing and responding to hypoxia and pH fluctuation, the nucleolus also plays a role in responding to redox stress. Translocation of NPM1 is central to the response to some nucleolar stressors; however, the causes of this translocation are unclear [35]. When NPM1 is S-glutathionylated after experiencing nucleolar oxidation, it triggers the dissociation of NPM1 from nucleolar nucleic acids [36]. NPM1 translocates from the nucleolus to nucleoplasm, where it sequesters HDM2, causing activation of p53. Therefore, the translocation of NPM1 to the nucleoplasm is required for stress-induced activation of p53. This is confirmed by the observations in the mutant NPM1 model where NPM1 is unable to be glutathionylated and remains in the nucleolus under nucleolar stress, thus preventing p53 activation [37].

Finally, in *Saccharomyces cerevisiae*, heat shock stress has leads to the disassembly of the nucleolus. Heat shock causes changes in gene expression, nucleolar morphology, and inhibition of rRNA synthesis [38]. Additionally, it has been found to cause many yeast nucleolar proteins, including the fibrillarin homolog Noplp, to relocate to the cytoplasm. Heat shock was found to inhibit protein import into the nucleus, thereby leading to the disassembly of the nucleolus. These findings clearly indicate the effects of heat shock stress on the anatomy of the nucleolus and rRNA transcription [39].

## Involvement of the nucleolus in DNA damage stress response

Response to DNA damage is a critical and highly regulated aspect of cellular biology. It ensures the dynamic and meticulous conservation of cell viability and genome fidelity. DNA damage, if left uncorrected, can result in mutations which can eventually lead to cell death or an aberrant cell survival that may lead to cancer [40]. A cell has an extensive network of proteins responsible for sensing and responding to DNA damage lesions, that include proteins responsible for apoptosis, DNA repair, and cell cycle arrest [41]. The tumor suppressor p53 plays a central role in DNA damage response. p53 is known to play a role in ribosome biogenesis and is involved in the regulation of pro-apoptotic functions of the DNA damage response as well as cell cycle arrest [42].

There are multiple mechanisms by which the cell responds to DNA damage. These include the base excision repair (BER) pathway and the nucleotide excision repair (NER) pathway, which includes the homology-directed recombination (HDR) and the non-homologous end-joining (NHEJ) pathway. BER is responsible primarily for removing small base lesions from the genome while the NER pathway is responsible for repairing bulky helix-distorting lesions. Several mechanisms exist to repair DNA double-strand breaks. Double-stranded break launches an intricate DNA damage response that includes detection of the damage, subsequent signaling, and DNA damage repair. The kinase, ATM (Ataxia telangiectasia mutated), transduces double-strand break recognition into activation of cell cycle checkpoints and repair mechanisms. Upon activation, ATM phosphorylates the histone variant H2AX [43]. Once this has occurred, there are two major pathways for correcting the double-strand break. These are the homology-directed recombination (HDR) and non-homologous end-joining (NHEJ) pathway [44]. Interestingly, several of the protein members of these pathways are found to localize in the nucleolus, making a compelling case for a role of the nucleolus in DNA damage repair.

As mentioned previously, NPM1 is a nucleolar phosphoprotein that acts as an endoribonuclease for maturing rRNA transcripts [45]. Additionally, studies indicate that NPM1 plays a role in DNA damage repair. During DNA damage, NPM1 accumulates at double-strand DNA breaks and recruits additional proteins, such as APE1 and FEN1, which are involved in BER, to the nucleolus [46]. NPM1’s interaction with apurinic/apyrimidinic endonuclease 1 (APE1) [47] is crucial to NPM1’s role in the BER pathway. The role of the NPM1-APE1 interaction has yet to be fully elucidated; however, studies have found that it regulates multiple cellular functions, including ribosome biogenesis and genomic stability in the nucleoplasm and nucleus [48]. Additionally, the binding of NPM1 to APE1 regulates the activity of APE1 in DNA repair [48]. Furthermore, in cells knocked down for NPM1, BER activity is impaired due to the inability of the nucleolus to retain BER factors. This indicates that NPM1 plays a role in promoting efficient BER [46]. NPM1 has also been found to interact with the tumor suppressor protein Rb leading to transcriptional activation of several DNA repair proteins involved in the NER pathway [49] (Table 2).

Like NPM1, nucleolin (NCL) is a multifunctional chaperone and nucleolar protein. NCL is known to play a role in DNA double-strand break (DBS) damage repair. NCL is not only recruited to DSB-induced foci, but also interacts with proteins involved in the DNA DSB response such as phosphorylated histone H2AX, as well as both HDR and NHEJ factors including RPA34, NBS1, and XRCC6, respectively [50]. Cells knocked down for nucleolin show increased radiosensitivity [51] and reduced capacity to relygate double-strand breaks [52] (Table 2).

The Werner syndrome RecQ-like helicase (WRN) is a protein implicated in Werner syndrome (an autosomal recessive disease characterized by a predisposition to cancer and premature aging) [53]. WRN has been found to play a role in a number of different DNA repair pathways including DNA DSB repair as well as telomere maintenance [54]. WRN has been found to localize to the nucleolus where it interacts with polymerase delta, a major DNA polymerase required for chromosomal DNA replication [55], indicating a direct control of WRN on DNA replication. In addition, inhibition of rRNA transcription results in the release of WRN from the nucleolus into the nucleoplasm. WRN is found to co-immunoprecipitate with Pol I and cells knocked down for WRN show decreased levels of rRNA transcription resulting in a reduction in the levels of 18S and 28S ribosomal subunits [56]. Thus WRN plays multiple roles in replication, DNA damage repair, and rRNA transcription through its presence in the nucleolus (Table 2).

Bloom syndrome RecQ-like helicase (BLM) is another helicase in the same family as WRN that is involved in DNA double-strand break repair pathways such as NHEJ, HR, and the amendment of stalled replication forks [57]. Like WRN, it is known for its role in DNA replication and repair, as well as telomere maintenance. Mutations in BLM can also result in severe growth defects and a predisposition to cancer and other diseases [57]. However, recent studies indicate a novel role of BLM in the nucleolus that is unique from its roles in preserving genome integrity. Similar to WRN, BLM is a confirmed nucleolar protein [58] and is found to interact with Pol I through IP studies [59]. Functionally, BLM has been found to be involved in pre-rRNA transcription via its helicase activity [59]. Pulse-chase analysis in BLM-deficient cells found a reduction in rDNA transcription [59]. This reduction in pre-rRNA transcription results in a decrease in mature 18S and 28S rRNA, and can trigger a cell stress response and apoptosis [60] (Table 2).

It is critical to note the fact that rDNA repeats are statistically more probable for susceptible to DNA damage. Needless to say, that these are one of the most highly transcribed genetic loci in the eukaryotic genome. Thus discord between transcription and replication in rDNA is particularly common [61]. When a double-strand DNA break occurs within the rDNA, the rDNA moves from the interior of the nucleolus to anchoring points at the periphery to form nucleolar caps allowing repair factors access to the rDNA to execute repair [62]. This rearrangement is coupled with an ATM-dependent inhibition of transcription by RNA Pol I [63]. The majority of double-strand breaks in rDNA are repaired via the NHEJ pathway [64]. Repair factor, 53BP1 is found to localize to nucleolar caps and associated with double-strand breaks, however, other repair factors such as ku80 and XRCC4 have not been found to localize to the nucleolar caps [63]. In addition to the NHEJ repair pathway, the HDR pathway has also been found to play a role in rDNA double-strand break repair in some instances. Nucleolar caps are reported to positively stain for rDNA and the DNA damage marker γH2AX, as well as HDR mediators such as BRCA1, Rad51, and RPA2 [63]. These finding indicate the importance of both, NHEJ and HDR, in rDNA repair and suggest that rDNA damage that cannot be repaired via the NHEJ pathway is reorganized and repaired by the HDR pathway at the nucleolar caps [65]. rDNA damage repair that occurs with the NHEJ pathway tend to be more error prone, while the HRD repair pathway is better able to preserve rDNA stability but can also result in rDNA copy number alterations [66]. Additionally, rDNA gene clusters have been identified as recombinatorial hot spots in human cancer. Over half of lung and colorectal carcinoma patients have some level of rDNA rearrangement [67], and genomic rearrangements of rDNA, such as insertions and amplifications are frequently observed in Hodgkin’s lymphoma [68]. These finding illustrate the possibility that genomic rearrangement, specifically in the rDNA, is selected for during tumor development and that the preservation of rDNA stability could further elucidate tumor suppressive mechanisms [69].

**Fig. 1:**
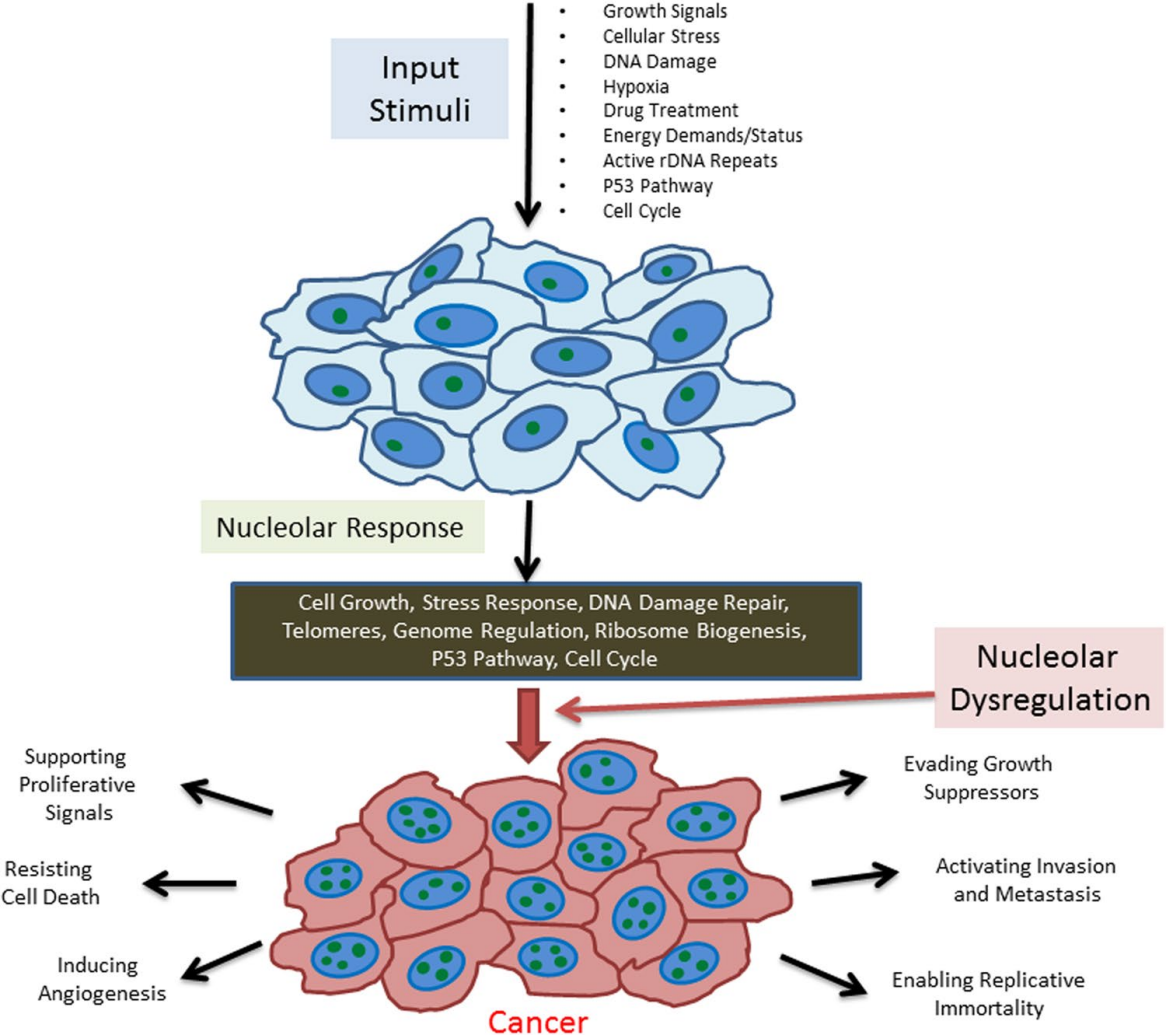
The nucleolus influences multiple activities critical to cancer progression. The nucleolus responds to several different types of cellular stimuli to regulate ribosome biogenesis and stress responses. The nucleolus coordinates multiple signaling pathways by evaluating the overall well-being of the cell. It also responds to alterations in the cell’s overall status to drive ribosome biogenesis, DNA damage repair, and cell cycle regulation among many other cellular responses. When the nucleolus becomes dysregulated, the resulting disruption in cellular processes can drive tumorigenesis and progression reflected as an increase in nucleolar number and/or size and a metabolically active state that drives key attributes of cancer progression

DNA damage is a cellular stress or survival challenge. As summarized before, it is becoming very clear that the role of the nucleolus in stress response is much broader and also applicable to different other types of cellular stressors (Fig. 1).

## Significant connection of the nucleolus and ribosomal proteins to cancer (Table 1)

In the late eighteenth century, an Italian pathologist, Giuseppe Pianese, described enlarged and more numerous nucleoli as one of the first noted markers of cancer [70]. Until recent time, pathologists visualized nucleoli using AgNOR staining for aiding clinical comments [71]. Several histopathological studies have reported correlative association of AgNOR staining with tumor stage, grade and aggressiveness [13, 72]. However, whether this increase in nucleolar size and number is a cause or an effect of tumorigenesis remains unclear. The prevailing thought is consistent with the commonsense knowledge that a tumor is a rapidly proliferating tissue and thus it has high demand for protein production which is met by increased ribosomal number. However, more in-depth studies have revealed that the nucleolus may be involved in multiple aspects of oncogenesis and tumor progression.

The role of the nucleolus in oncogenesis has been noted as a sequela of other disease pathologies. Dyskeratosis congenita is a disease characterized by abnormal skin pigmentation, nail dystrophy, leucoplakia, and bone marrow failure and is associated with an increased risk of cancer [73]. This disease is caused by a mutation in the DKC1 gene which codes for the protein dyskerin, a nucleolar protein that is responsible for the posttranscriptional pseudouridylation of rRNA which is crucial for proper rRNA folding and eventual ribosome biogenesis [74]. Dyskerin is also a component of the telomere enzyme complex that is responsible for maintaining telomeres and immortality, a prerequisite to carcinogenesis [75].

Another disease pathology associated with the nucleolus is Diamond Blackfan anemia, a rare genetic disorder that affects the ability of the bone marrow to produce red blood cells. Seventy percent of cases of Diamond Blackfan anemia are caused by mutations in ribosomal protein genes [76]. Specifically, mutations in the small ribosomal subunit protein S19 are associated with an increased susceptibility to hematopoietic malignancies [77]. Myelodysplastic syndrome (MDS) is a disease characterized by peripheral cytopenias, hypercellular bone marrow, and an increased rate of mortality resulting from the development and progression of acute myeloid leukemia [78]. In MDS cells, ribosomal protein L23 (RPL23) functions as a negative regulator of cell apoptosis. Increased expression of RPL23 was found to be an independent prognostic predictor. Furthermore, compared to patients with normal levels of RPL23, patients with high levels of RPL23 were significantly more likely to develop acute myeloid leukemia and a correspondingly reduced survival rate [79].

In addition to alterations in nucleolar proteins that can lead to a predisposition for cancer development, alterations in ribosomal DNA copy number are observed in cancers. The most common alterations to the rDNA copy number in cancers are amplification of 5S rDNA repeats and a reduction or loss of 45S rDNA [80]. Cancer genomes with lower 45S rDNA copies show evidence of mTOR hyperactivity. PTEN, a negative regulator of the mTOR pathway, plays a crucial role in genome stability. In a Pten^−/−^ mouse model for leukemia, cancer stem cells were found to have lower rDNA copy number than normal tissue, even though this model has increased proliferation, rRNA production, and protein synthesis. This observed reduction in copy number is also associated with hypersensitivity to DNA damage. As such, 45S rDNA copy number reduction in cancer is associated with mTOR activation and may prove to be a simple way to determine if a cancer will be susceptible to DNA-damaging treatments [81].

**Fig. 2:**
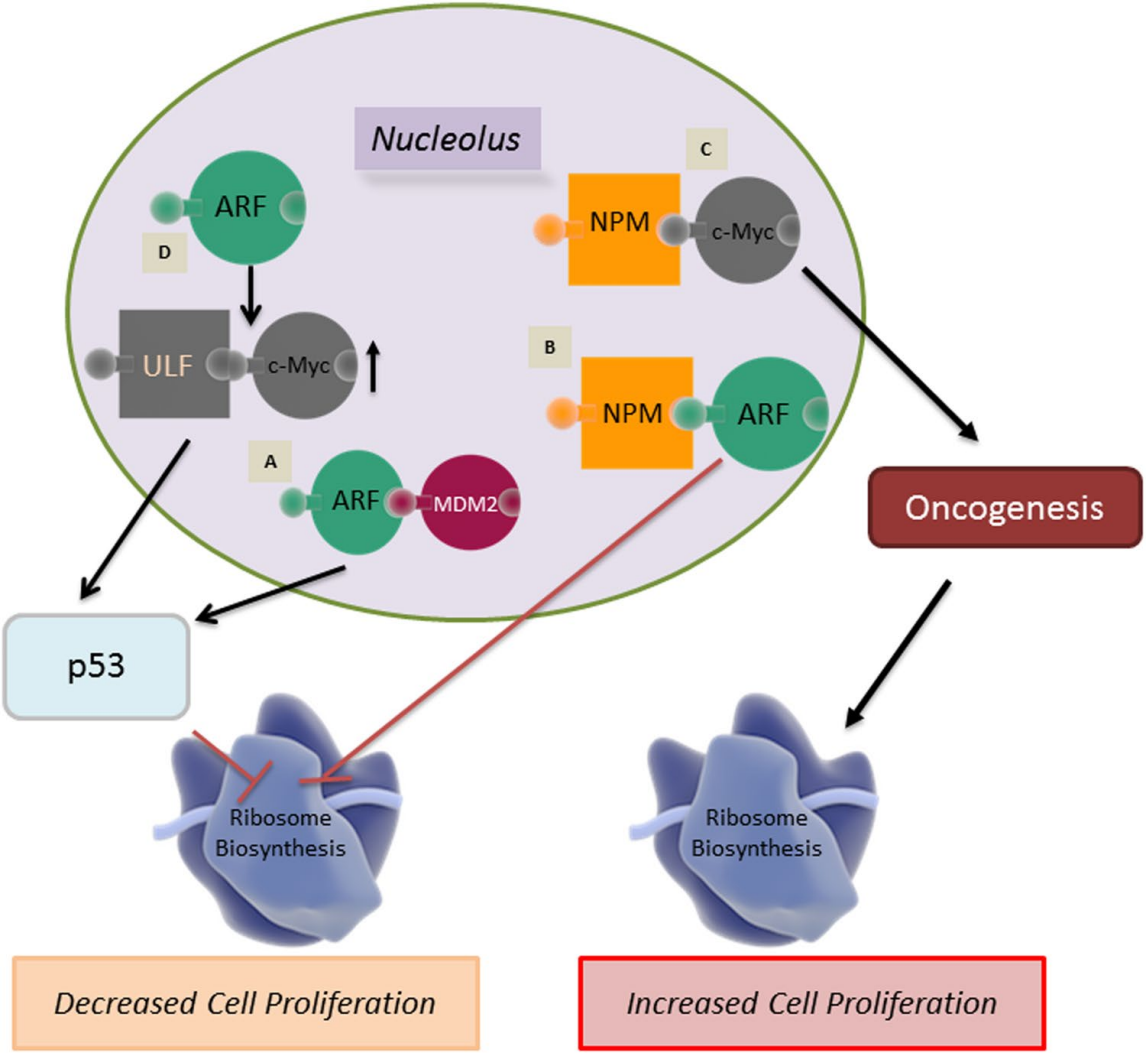
Role of nucleolus in controlling proliferation of cancer cells. ARF and NPM1 are key nucleolar proteins that regulate proliferation through tumor suppressor p53 and oncogene c-Myc. **A** The binding of ARF to MDM2 allows the complex to translocate to the nucleolus allowing for the activation of the p53 pathway. **B** Binding of NPM1 and nucleolar ARF acts as a checkpoint for ribosome biogenesis. **C** Binding of NPM1 to c-Myc regulates hyperproliferation and transformation. **D** Role of c-Myc is context dependent. An increase in nucleolar c-Myc causes the stabilization of ARF resulting in ULF binding to c-Myc leading to the activation of the p53 pathway

One of the better elucidated connections of the nucleolus to cancer, specifically oncogenesis, is the role of nucleolar ARF (Fig. 2). The protein ARF is formed as a result of an alternative reading frame for the same gene that codes for the Rb protein regulator p16INK4a. ARF is the second most commonly lost protein in cancer after p53 and contains a nucleolar localization signal (NoLS) [82]. In normal tissue samples, levels of ARF are usually extremely low; however, upon stimulation from various oncogenic signals, such as Ras and Myc, levels of ARF are found to be increased significantly in coordination with nucleolar localization [83]. This accretion of nucleolar ARF causes an inhibition of cell cycle progression by directly interacting with the *p53*-ubiquitin ligase Mdm2 (murine double minute 2) oncoprotein. The binding of ARF to Mdm2 results in a conformational change in Mdm2, allowing the ARF-Mdm2 complex to translocate to the nucleolus [84, 85]. This leads to the stabilization of p53 tumor suppressor in the nucleoplasm, thereby allowing p53 to induce the expression of downstream-negative regulators of proliferation [83, 86]. Additionally nucleolar ARF has been found to inhibit the maturation of rRNA, which has been found to reduce both rRNA synthesis, as well as protein translation, resulting in further prohibition of cell cycle progression [87].

NPM1 has been found to physically interact with ARF in the nucleolus [88]. This ARF-NPM1 interaction negatively regulates rRNA processing within the nucleolus, thereby acting as a checkpoint for ribosome biogenesis [45] (Fig. 2). Alternatively, it has been shown that the shuttling of NPM1 between the nucleolus and the cytoplasm is crucial to its ability to promote cell proliferation [89]. The interaction of ARF with NPM1 to prevent ribosome biogenesis further highlights the key role of the nucleolus as a domain that controls critical molecular steps and pathways in cancer. Without this ARF checkpoint, ribosome biogenesis would proceed unchecked, promoting tumorigenesis.

ARF-mediated p53-dependent pathway activation results primarily from oncogenic stress, which is known to drive Pol I transcription [90]; however, c-Myc also plays an important role in mediating oncogenic stress [91]. c-Myc is a well-studied transcription factor and an oncogene that is known to regulate several different genes that modulate cellular differentiation, proliferation and growth [92]. Additionally, evidences support c-Myc’s direct role in ribosome biogenesis due to the fact that c-Myc is an activator of all three RNA polymerases [93–95]. NPM1 directly interacts with c-Myc and controls hyperproliferation and transformation [96]. In addition, NPM1 is required for the localization of c-Myc to the nucleolus where it is involved in the initiation of rRNA synthesis. Therefore, a combination of modulated nucleolar functionality as a sequestration domain and rDNA transcription regulation by c-Myc are necessary for c-Myc to act as a potential oncogene [92]. Alternatively, in a functionally normal nucleolus, the overexpression of c-Myc triggers the suppression of rRNA synthesis by activating ARF stabilization and subsequent activation of the p53 pathway [97]. When this occurs, c-Myc forms a complex with ULF (ubiquitin ligase of ARF) which prevents ULF from ubiquitinating ARF, thereby preventing degradation of ARF, resulting in increased nucleolar ARF levels (Fig. 2) [98]. Two separate p53-dependent pathways can be activated simultaneously to neutralize the results of c-Myc oncogenic activity. In one pathway, ARF mediates the previously mentioned ARF–Mdm2–p53 axis [99]. In another pathway RPs, create an RP–Mdm2–p53 axis [85, 100]. The activation of any one of these pathways can induce cell cycle arrest, apoptosis or autophagy.

The nucleolar N-terminal truncated isoform of netrin 1 (ΔN-netrin-1) lends excellent support to the concept that increased ribosomal numbers may be required to drive malignant transformation. Netrins are a class of proteins normally involved in cell migration and axon guidance during development [101]. The ΔN-netrin-1 isoform binds to components of Pol I in cancer cells [102], thereby driving rDNA transcription and pre-rRNA processing, resulting in an increase in the number of mature ribosomes in tumor cells, promoting the malignant phenotype [102].

Nucleolin (NCL) is another important nucleolar protein that plays a role in cancer development and metastasis. This well-characterized protein is known for its role in ribosome biogenesis; however, NCL does not localize exclusively to the nucleolus, and can also be found in the cytoplasm and on cell surface membranes. Various ligands are able to bind to cell surface NCL and affect the physiological functions of the cell [103]. Interaction of endostatin with NCL at the surface of endothelial cells potentiates an antiangiogenic response and inhibition of tumor growth. Disruption of this interaction using a neutralizing antibody (or siRNA against NCL) nullifies the antiangiogenic effects of endostatin. Interestingly, when NCL localizes to the nucleus endostatin is able to inhibit phosphorylation of NCL, thereby inhibiting its role in cell proliferation [104] (Table 2).

Finally, crucial to the nucleolus’ ability to sense and respond to cellular stress are ribosomal proteins (r-proteins). There have been many instances of overexpressed ribosomal proteins across many different types of cancers. For example, RPLP0, RPLP1 and RPLP2 are found to be upregulated at the RNA level in patients with gynecologic tumors, and this upregulation positively correlated with the presence of lymph metastasis in ovarian cancers [105]. Additionally, the overexpression of ribosomal protein RPS11 and RPS20, together and individually, have been found to be a consistent predictor of poor survival outcome in patients with glioblastoma [106]. Several different ribosomal proteins have also been studied in vitro. In the colorectal cell lines, HCT116 and HT-29, knockdown of RPS24 was found to inhibit cell migration and proliferation [107]. Alternatively, overexpression of RPL19 in the breast cancer cell line MCF7 was found to make cells more susceptible to stress-induced cell death [108].

Several r-proteins play a role in regulating changes in p53 levels independent of DNA damage, as well as maintaining nucleolar structure. Most notably among these are RPL5 and RPL11, which have been established as critical mediators of the activation of p53 during ribosomal stress [109]. These two ribosomal proteins bind to HDM2, thereby preventing the degradation of p53. In a recent study, Nicolas et al. observed that not only are RPL5 and RPL11 the strongest contributors to the maintenance of nucleolar structure but are also required for the activation of p53 when other ribosomal protein levels are reduced [110]. This is likely achieved by the interaction of these two proteins (singularly or in concert) with HDM2 to squelch away HDM2 leading to stabilization of p53.

## Emerging importance of rRNA modifications in cancer

There are many additional activities that are regulated through the nucleolus and may have pivotal roles in carcinogenesis and/or tumor progression. The nucleolus serves as an important hub of rRNA secondary modifications. This central involvement of the nucleolus in posttranscriptional modifications has far reaching impacts on ribosome structure and function. Studies have detailed the importance of secondary modifications on rRNA stemming from the two most abundant modifications, most of which classify as ribose methylations (2′-O-Me) or pseudouridines (ψ) which tend to cluster in regions of the rRNA assumed to be the most functionally important [111]. These rRNA modifications lend their importance in mediating ribosome function due to the fact that they tend to cluster in regions of the rRNA assumed to be the most functionally important, including the decoding site and peptidyl transfer center [112, 113]. Furthermore, ribosomes are also comprised of many ribosome-associated proteins which seem to play a critical role in amending ribosome structure and translational efficiency [114, 115]. The importance and function of these accessory proteins and rRNA modifications that occur in the nucleolus, are poorly understood. However, studies are beginning to shed light on the importance of secondary modifications of rRNA and ribosomal proteins for functional and structural diversity [116–118]. Two classes of small non-coding RNAs termed as small nucleolar RNAs (snoRNAs) act as guides for posttranscriptional synthesis of the 2′-O-Me and ψ on rRNA. snoRNAs are grouped according to their conserved sequence elements and associate with a core of snoRNP proteins that direct modification on the rRNA. Box C/D snoRNAs associate with fibrillarin, Nop56p, Nop58p, and 15.5KDa to guide2′-O-methylation; whereas box H/ACA snoRNAs form a complex with dyskerin/Cpf5p, Gar1p, Nhp2p, and Nop10b to guide pseudouridylation [119]. Particularly in the context of cancer, studies have consistently demonstrated that snoRNAs play an important role in disease progression. Early studies in breast cancer detailed the functional relevance between enhanced rRNA methylation and ribosome biogenesis as it relates to disease progression [14]. More recently, detailed analysis of both, mouse and human breast cancer models were reported to have increased snoRNA and fibrillarin expression. Furthermore, knockdown of snoRNPs, fibrillarin, NOP56, or NOP58 yielded significant detrimental effects on tumorigenicity, further solidifying the importance of snoRNA in cancer progression [120–123].

Dyskerin has divergent functions; it plays a role in telomerase activity and pseudouridylation of rRNA, as well as acts as H/ACA snoRNP and is an important player in mediating rRNA modifications. Dyskerin expression and function has been shown to be associated with breast, lung, colon, and lymphoma tumor progression [74, 124–126]. Multiple studies involving dyskerin have underscored the importance of rRNA pseudouridylation in modulating ribosome translation to promote oncogenic properties of cells [124, 127, 128]. These studies collectively accentuate the importance of not only fibrillarin and dyskerin, but rRNA modifications, as key drivers of cancer progression as a result of changes in the ribosome structure and function. Nonetheless, the exact functional impact of these modifications remains unknown.

## The nucleolus as a drug target for cancer

Aberrant regulation of Pol I transcription and ribosome biogenesis is pervasive in many different types of cancer [13]. Unlike many ribosomopathies, the altered rDNA transcription activity that is often seen in cancers is not due to amplifications or mutations in the Pol I transcription apparatus. Rather, elevation of Pol I transcription during cancer is typically the result of over-activation of an oncogenic signaling pathway or the lack of inhibitory effect of signaling driven by tumor suppressors [129]. Currently, it is unclear if accelerated rDNA transcription in cancer is sufficient to initiate malignant transformation. However, there are interesting associations of accelerated Pol I activity with oncogenesis. c-Myc, a well-documented player in cancer development and progression, has been shown to be capable of increasing Pol I activity, thereby increasing ribosome biogenesis. Activation of c-Myc in fibroblasts or human B-cells leads to enrichment of Pol I transcription factor SL1 at the rDNA promoter and an increase in pre-rRNA synthesis. c-Myc also enhances ribosome biogenesis indirectly by upregulating the expression of ribosomal protein, UBF, and other proteins involved in rRNA maturation [130]. Unfortunately, it has not yet been possible to attribute this role of c-Myc to malignant transformation due to the fact that c-Myc plays many other roles in cell growth and tumor progression in addition to its role in ribosome biogenesis. This does not, however, eliminate the possibility that tumor cells can become ‘addicted’ to ribosome biogenesis. This causes cells to become vulnerable to selective inhibition of rRNA synthesis. Many of the chemotherapeutic agents used in the clinic today, including cisplatin, doxorubicin and flavopiridol, mediate their therapeutic effects, at least in part, through the disruption of ribosome biogenesis [131–133].

Recent advances have led to the development of several new compounds that selectively block ribosome biogenesis. One of the first such drugs to achieve this effectively was the small molecule CX-5461, developed by Cylene Pharmaceuticals, which blocks SL1 from binding to the rDNA promoter, effectively inhibiting Pol I transcription [134]. Using this compound, the same group then demonstrated that both an increase in rDNA transcription as well as an enacted nucleolus is required for oncogenesis in hematologic tumor cells [135]. Additionally, they used CX-5461 to target Pol I activity in a therapeutic capacity to treat tumors in mouse models of lymphoma and leukemia by activating p53-mediated apoptosis while leaving normal cells unharmed [135]. This p53-induced apoptotic death occurred within hours of treatment in tumor cells, caused by resulting nucleolar stress and was irrespective of any changes in ribosome or protein production. This latter finding is particularly relevant because it indicates the importance of nucleolar integrity in the survival of tumor cells regardless of the role the nucleolus plays in regulating ribosome biogenesis, protein production, and cell growth. These findings further support the key role that the nucleolus plays in cancer cells, independent from its role in ribosome biogenesis [136].

Another inhibitor of Pol I that has recently been developed is BMH-21. This small molecule binds to the rDNA promoter and causes degradation of RPA194 (Pol I catalytic subunit), thereby inhibiting Pol I activity. This compound differs from others in that it induces nucleolar stress without activating the cell’s DNA damage response signaling and repair [137]. Additional studies indicate that both polymerase subunits and Pol I preinitiation factors are required for BMH-21 to effectively degrade RPA194. Furthermore BMH-21 was found to inhibit transcriptional elongation by Pol I, further inhibiting ribosome biogenesis. Finally, genetic studies have found that BMH-21 hypersensitivity can be induced in cells that have mutations that induce transcription elongation defects in Pol I [138].

In addition to these newly developed chemotherapeutic agents that will target the nucleolus and ribosome biogenesis specifically, it has also come to light that several commonly used standard of care chemotherapeutics also target the nucleolus and thereby target ribosome biogenesis. A study by Burger et al. found that more than half of the 36 chemotherapeutic agents screened affected ribosome biogenesis at some level of the process [139]. Two of the most commonly used chemotherapeutics in research, doxorubicin and cisplatin, were both found to alter ribosome biogenesis. Doxorubicin is an anthracycline antibiotic that acts as a DNA intercalator and inhibitor of topoisomerase II. Treatment with doxorubicin leads to a decrease in rDNA transcription and causes changes in nucleolar morphology where nucleoli become smaller and form a ring-like shape [140]. Additionally, cisplatin, a platinum compound that acts as a DNA cross-linking agent, causes cross-linking between DNA and UBF, thereby preventing UBF from binding to rDNA and promoting rDNA transcription. Treatment with cisplatin also causes the redistribution of important ribosome biogenesis factors such as UBF, TAFs and Pol I within the nucleolus further inhibiting ribosome biogenesis [141, 142].

These recent advancements in selective small molecule inhibitors of Pol I provide an exciting opportunity to investigate the unbridled relationship between Pol I transcription and cancer. The fact that these inhibitors can selectively kill cancer cells while leaving healthy cells unharmed provides insight into an exciting new class of antineoplastic drugs that may significantly advance cancer treatment.

## Conclusions

The nucleolus is widely denoted as the primary site for ribosome biogenesis, and in that context, RNA Pol I is a very important target that has been widely investigated. Advances in proteomics, including the Human Protein Atlas, have provided novel insight into the nucleolus. Rapidly evolving microscopic techniques have brought clarity to the spatio-temporal context for individual proteins. This has allowed revelation of compartmentalization of proteins and sub-cellular venues for several novel protein–protein and protein–RNA interactions. Contextual contributions of the nucleolar presence and interactions of these proteins and RNA species remain to be elucidated. Additionally, it has yet to be elucidated whether alterations seen in the nucleolus are driving factors in carcinogenesis or more passive events. Due to the fact that many ribosomopathies lead to a predisposition to cancer, it does seem likely that the nucleolus and the process of ribosome biogenesis are one of many drivers in tumorigenesis.

The link between nucleolar alterations, and, therefore, enhanced ribosome biogenesis, has been well documented in various pathologies of chronic inflammatory diseases such as chronic liver disease, ulcerative disease of the colon, and chronic pancreatitis, all of which lead to enhanced cancer risks. Cancer cells become “pre-conditioned”, during a transitional state, to rely on the alterations in ribosome biogenesis or on differentially modified ribosomes that translate a unique subset of mRNAs that cancer cells require for survival and progression [14, 115]. At this stage, cancer cells become “addicted” to changes in ribosome biogenesis in coordination with specialized ribosomes, which are needed to translate vital subsets of mRNAs to maintain survival and potentiate cancer progression [143, 144]. The vulnerability of cancer cells lies in their need to have unique requirements in ribosome biogenesis in combination with “specialized” ribosomes to carry out vital functions for tumor cell maintenance and progression [143, 145]. As previously discussed, current therapeutic strategies aim to target the reliance of cancer cells on altered ribosome biogenesis. Furthermore, therapies that could target ribosomes with unique secondary modifications may be of great interest. More recently, studies have begun to detail distinct populations of ribosomes that are inherent to cancer cells; consequently, if inhibition of ribosome function can be targeted against these “specialized” ribosomes, this may prove to be a viable option for highly specific targeted therapy to cancer cells [143, 145–147].

Given the sizable number of nucleolar proteins that have yet to be characterized, we predict that the role of the nucleolus in both normal and cancer biology will continue to evolve. We forecast that as new data are uncovered regarding the nucleolus, additional mechanistic connections of the nucleolus to cancer progression will be elucidated. Insulin is involved in multiple pathologies and has emerged as one such regulator. Insulin receptor substrate 1 has been shown to translocate to the nucleolus and bind to UBF1. Additionally, type I insulin-like growth factor receptor signaling can increase the phosphorylation of UBF1 leading to an increase in transcription from the rDNA promoter [148]. Furthermore, IGF-1 is shown to activate Pol I transcription by increasing SL1 occupancy on rDNA promoters [149]. Considering the central importance of the nucleolus, there possibly exist many more modulators of nucleolar functions and ribosome biogenesis. However, the identity and contributions of signaling factors and ligands, which play critical roles in modulating nucleolar functions and ribosome biogenesis, in multiple pathologies including cancer, remain to be described.

These details are of utmost importance to understanding functional contributions of these biomolecules to both normal and cancer biology. It certainly can be anticipated that studies in these directions will open up the nucleolus as a drug discovery platform and gateway for novel cancer therapeutic strategies.

In summary, the nucleolus potentially holds keys to multiple activities that influence cancer progression (Fig. 1). Unraveling deeper details of nucleolar biology in the context of cancer may contribute to the development of several novel cancer drug targets.
